# Non-invasive continuous blood pressure monitoring (ClearSight™ system) during shoulder surgery in the beach chair position: a prospective self-controlled study

**DOI:** 10.1186/s12871-020-01185-6

**Published:** 2020-10-24

**Authors:** Konrad Chachula, Florian Lieb, Florian Hess, Joellen Welter, Nicole Graf, Alexander Dullenkopf

**Affiliations:** 1 Institute for Anesthesia and Intensive Care Medicine, Spital Thurgau Frauenfeld, Frauenfeld, Switzerland; 2 Clinic for Orthopedic Surgery and Traumatology, Spital Thurgau Frauenfeld, Frauenfeld, Switzerland; 3 Graf biostatistics, Winterthur, Switzerland

**Keywords:** Monitoring, blood pressure, Patient monitoring, General anesthesia, Beach chair position

## Abstract

**Background:**

The beach chair position that is commonly used in shoulder surgery is associated with relative hypovolemia, which leads to a reduction in arterial blood pressure. The effects of patient positioning on the accuracy of non-invasive continuous blood pressure monitoring with the ClearSight™ system (CS-BP; Edwards Lifesciences, Irvine CA, USA) have not been studied extensively. Our research aim was to assess agreement levels between CS-BP measurements with traditional blood pressure monitoring techniques.

**Methods:**

For this prospective self-controlled study, we included 20 consecutively treated adult patients undergoing elective shoulder surgery in the beach chair position. We performed Bland-Altman analyses to determine agreement levels between blood pressure values from CS-BP and standard non-invasive (NIBP) methods. Perioperative measurements were done in both the supine (as reference) and beach chair surgical positions. Additionally, we compared invasive blood pressure (IBP) measurements with both the non-invasive methods (CS-BP and NIBP) in a sub-group of patients (*n* = 10) who required arterial blood pressure monitoring.

**Results:**

We analyzed 229 data points (116 supine, 113 beach chair) from the entire cohort; per patient measurements were based on surgical length (range 3–9 supine, 2–10 beach chair). The mean difference (±SD; 95% limits of agreement) in the mean arterial pressure (MAP) between CS-BP and NIBP was − 0.9 (±11.0; − 24.0–22.2) in the beach chair position and − 4.9 mmHg (±11.8; − 28.0–18.2) when supine. In the sub-group, the difference between CS-BP and IBP in the beach chair position was − 1.6 mmHg (±16.0; − 32.9–29.7) and − 2.8 mmHg (±15.3; − 32.8–27.1) in the supine position. Between NIBP and IBP, we detected a difference of 3.0 mmHg (±9.1; − 20.8–14.7) in the beach chair position, and 4.6 mmHg (±13.3; − 21.4–30.6) in the supine position.

**Conclusions:**

We found clinically acceptable mean differences in MAP measurements between the ClearSight™ and non-invasive oscillometric blood pressure systems when patients were in either the supine or beach chair position. For all comparisons of the monitoring systems and surgical positions, the standard deviations and limits of agreement were wide.

**Trial registration:**

This study was prospectively registered at the German Clinical Trial Register (www.DRKS.de; DRKS00013773). Registered 26/01/2018.

## Background

Systolic and mean arterial blood pressure tend to be lower in the sitting than the supine position because of relative hypovolemia and reduced cardiac pre-load [1, 2]. In orthopedic surgery, many shoulder operations are performed with patients in the beach chair position. This position provides the surgeon with increased accessibility to the target area, is intended to prevent high blood pressure to reduce blood loss, and improves arthroscopic visibility. While keeping patients at moderate levels of hypotension is often well tolerated and can be considered safe [3, 4], the margin of safety may be small. Recently published data convincingly correlated intraoperative arterial hypotension with unfavorable outcomes [5–7].

It is well known that blood pressure varies with body positon, being lower in the sitting compared to the supine position [1, 8, 9]. This blood pressure drop is increased when bringing anesthetized patients in the sitting or beach chair position [2]. The accuracy of blood pressure measurement depends on patient positioning [1]. Innovative non-invasive continuous ABP monitoring technologies are currently available [10–12], and seem to contribute to hemodynamic stability in settings, such as general anesthesia in orthopedic patients [13]. One such device, the ClearSight™ system (Edwards Lifesciences, Irvine CA, USA), monitors beat-to-beat blood pressure using the volume clamp or vascular unloading method and assesses cardiac output by pulse contour analysis with an inflatable finger cuff [10, 14]. While data are available about its use in the general population and subgroups of obese [15], cardiac [16, 17] and orthopedic patients [18], little is known about patients undergoing surgery in the beach chair position.

The primary aim of this prospective self-controlled study carried out under clinical conditions was to assess the level of agreement between two non-invasive BP methods (continuous ClearSight™ (CS-BP) and intermittent NIBP) when shoulder surgery patients were in the beach chair position, and also while they were in the supine position. As secondary aim, we compared non-invasive continuous monitoring with CS-BP to invasive continuous arterial blood pressure monitoring in a subgroup of patients who required additional arterial monitoring. These comparisons were made while the patients were in the beach chair and supine positions. This study was not, however, a formal validation of the device in the beach chair position. Likewise, our study was not evaluating the safety of the absolute BP values measured in different positions. Our institution’s standard of care dictates that we maintain a moderate level of hypotension in patients undergoing this type of surgery; therefore, such controlled circumstances would be unsuitable for a safety study. We hypothesized that the volume clamp or vascular unloading technology provided acceptably low bias compared to conventional blood pressure monitoring, irrespective of patient positioning.

## Methods

This prospective, self-controlled study was performed between January and May 2018 at a cantonal level hospital in eastern Switzerland. After receiving approval of our research protocol by the Ethics Committee of Eastern Switzerland (EKOS; 2017–01680), written informed consent of eligible patients was obtained. The study was recorded with the German Clinical Trial Register (www.DRKS.de; DRKS00013773).

**Fig. 1:**
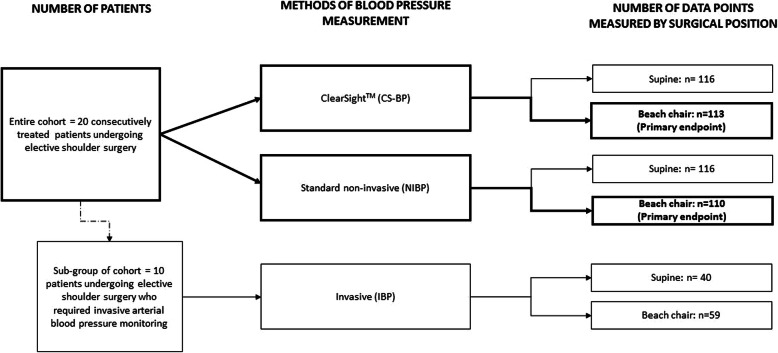
Diagram illustrating study design and total data points collected (*n* = 229)

We assessed for inclusion consecutively treated patients undergoing arthroscopic shoulder surgery performed in the beach chair position. The inclusion criteria were: 1) adults aged 18 years or older, 2) surgery to be performed under general anesthesia, 3) non acute-trauma (elective) procedures, and 4) patients with a body mass index of > 20 and < 35 kg/m^2^. In a subgroup of patients, the additional inclusion criterion was a need for invasive arterial blood pressure monitoring, which was determined by the treating anesthetist who was not involved in the study (Fig. 1). Patients were excluded if they 1) they were hemodynamically instable prior to anesthesia induction or revealed relevant arrhythmia, 2) they had any of the following co-morbidities: severe vascular disease, peripheral vascular pathology, Raynaud syndrome, severe edema of the fingers or hands; 2) were participating in another study; or 3) were pregnant. No patient was enrolled in this study twice.

### Anesthesia management

All general anesthetics were performed according to institutional standards, and all therapeutic decisions were based exclusively on the results of the standard monitoring. After standard monitoring was applied and before anesthesia induction, patients received an interscalene block on the operated arm, when clinically indicated. General anesthesia was induced by propofol target-controlled infusion (Schnider model, 4–6 μg/ml effect site concentration) and i.v. fentanyl (approx. 1.5 to 3 μg/kg). Tracheal intubation was facilitated by administration of atracurium (0.5 mg/kg). Anesthesia was maintained with propofol, supplemented by fentanyl and/or continuous remifentanil infusion (based on the judgment of the attending anesthetist and bispectral index (BIS; Philips Healthcare; Zurich, Switzerland) values between 40 and 60). Patients were moved intraoperatively to the beach chair position. The responsible anesthetist handled fluid administration by Ringers acetate and blood pressure management by administering fluid, varying anesthetic depth or re-dosing opioids, and if necessary, the use of ephedrine. In general, the systolic blood pressure target was 100 mmHg (or maximum 30% lower than baseline) during surgery.

### Non-invasive continuous blood pressure measurement

The ClearSight™ system consists of an inflatable finger cuff that continuously assesses blood pressure and cardiac index (CS-CI). The CS-BP assessment technique is known as ‘vascular unloading technology’ or ‘the volume clamp method’ and has been described in detail elsewhere [10, 11]. Essentially, the method is based on a modified Penaz principle, which means to assess arterial pressure at the finger by analyzing the pressure required to keep the volume of a cuff around the finger constant despite the pulsating finger artery. CS-CI assessment is based on pulse contour analysis comparing the actual pulse curve to an extensive internal database [10]. The system repeatedly calibrates itself by analyzing the unloaded arterial blood volume. No external calibration is required once the system is zeroed to ambient pressure.

The appropriate ClearSight™ cuff size was determined based on the size of the patient’s index finger. As suggested by the manufacturer, the cuff was placed on the forefinger (index, middle or ring finger), and then the values were displayed on the ClearSight™ stand-alone monitor. The ClearSight™ system was zeroed to the ambiance at the level of the proximal end of the upper arm NIBP cuff. CS-BP was electronically recorded in 20-s intervals.

### Non-invasive intermittent oscillometric blood pressure measurement

Non-invasive intermittent oscillometric blood pressure measurements (MP 30, Philips Healthcare; Zurich, Switzerland) were obtained from the patient’s upper arm and recorded during the anesthetic in 2.5 to 5-min intervals depending on the patient’s hemodynamic situation. The size of the blood pressure cuff was selected based on the manufacturer’s recommendation.

### Invasive continuous arterial blood pressure measurement

Invasive continuous arterial blood pressure monitoring was performed after cannulation of the radial artery (Haemofix Exadyn Set; B. Braun; Melsungen, Germany) and displayed on the vital signs monitor (MP 30, Philips Healthcare; Zurich, Switzerland). The arterial monitoring system was zeroed to the ambiance at the level of the proximal end of the upper arm NIBP cuff.

### Data sources

The main study outcomes were mean values of ABP, heart rate, and cardiac index (CI). Different blood pressure monitors were used simultaneously on the same arm in every study participant, which was the non-operated arm. Recordings from the ClearSight™ system were done by an investigator who was not responsible for the anesthetic. We synchronized the clocks of the ClearSight™ system and the vital signs monitor before beginning with each patient’s measurements. Blood pressure was reported as systolic, mean (MAP), and diastolic. These were recorded in a supine resting position before and after anesthesia induction, after bringing the patient in the beach chair position and every 30 min thereafter, at the end of anesthesia in the supine position and then one last time after emergence from anesthesia while still in a supine position (see Fig. 1). During all measurements, the upper arm was positioned straight alongside the body with the forearm resting on an armrest. The exact time of oscillometric NIBP measurements was recorded. Heart rate from the vital signs monitor (MP 30, Philips Healthcare; Zurich, Switzerland), CS-BP, CS-CI and IBP were assessed immediately before initiating the NIBP measurement. CS-BP and CS-CI were recorded as the mean of the three previous recordings, i.e. the mean of a time span of 1 min. Additional study variables, such as basic patient and anesthesia data, were recorded on the patient’s anesthesia chart. At the end of general anesthesia, all patients were carefully assessed for skin damage under the finger cuff and any other complications from the measurement methods.

### Statistical methods

Mean values of ABP, heart rate, and cardiac index (CI) were obtained during supine positioning and were compared to mean values obtained in the beach chair position (Fig. 1) with exact Wilcoxon signed rank tests. Blood pressure values gathered by the different methods were compared using Bland-Altman analysis and took into account the use of multiple measurements [19]. CS-BP was compared to NIBP as the most frequently used clinical standard. Additionally, CS-BP and NIBP were compared to IBP as the reference method. The mean difference between NIBP and CS-BP was calculated by subtracting CS-BP values from NIBP values, and then calculating the weighted mean. The mean difference between the non-invasive methods (CS-BP and NIBP) and the invasive method (IBP) was calculated by subtracting the non-invasive values from the invasive values, and then taking the weighted mean. Differences in blood pressure measurements between the methods and according to patient positioning were tested with a weighted one-sample t-test. The correlation of differences in MAP values for CS-BP compared to NIBP, and IBP to heart rate and the cardiac index was calculated by Spearman’s rank correlation (in both supine and beach chair positions).

The MAP values with ≥10% deviation from the standard method were assessed for both CS-BP (to NIBP and IBP) and IBP (to NIBP) measurements. It was calculated how often an increase or decrease of ≥10% between subsequent MAP measurements of the standard method was accompanied by a change in CS-BP MAP in the same direction (i.e., either increase (> 0) or decrease (< 0) between subsequent measurements).

Reported *p*-values can be considered nominal and unadjusted for multiple testing. A power calculation was not performed since this was primarily a descriptive study. However, we estimated 20 patients with an average of 5 measurements (per patient and position) resulting in 100 data points would be sufficient when making comparisons using Bland-Altman plots [19]. All statistical analyses were performed using Microsoft Excel 2010 (Microsoft, Redmond, USA) and the statistical software package R version 3.3.3 (R Foundation for Statistical Computing, Vienna, Austria).

## Results

Twenty patients were included, and a total of 230 time points (117 in supine, 113 in beach chair position; 11.5 (± 3.5) time points per patient) were assessed. All patients underwent standard non-invasive blood pressure monitoring, and a subgroup of 10 patients also underwent invasive blood pressure monitoring (104 time points, 41 in supine, 63 in beach chair position). General patient and anesthesia data are shown in Table 1. Blood pressure values according to patient positioning are presented in Table 2. For all three assessment methods and in all blood pressure modalities (systolic, MAP, and diastolic), the ABP was lower in the beach chair position (all *p* < 0.001). Heart rate (*p* = 0.083) and CI (*p* = 0.388) did not differ. No complications from any of the methods were observed.

**Table 1:** General patient and anesthesia-related data

Parameter	Unit	Result
Age	Years	68.5 (54.3–81.3)
Height	m	1.7 (1.6–1.8)
Weight	kg	74.5 (65.8–85.5)
BMI	kg/m^2^	25.7 (22.9–31.9)
ASA	1–5	2 (2–3)
Gender	Female	11 (55%)
ClearSight™ sensor size	Small / Medium / Large	0 / 11 (55%) / 9 (45%)
Study period	min	156 (129–175)

Data are median (IQR) or n (%). *IQR* Interquartile range, *BMI* Body mass index, *ASA* American Association of Anesthesiologists physical status; study period = time from first to last recorded blood pressure measurement

**Table 2:** Mean values (standard deviation) of blood pressure (mm Hg), heart rate (min^− 1^), and cardiac index (l * min^− 1^ * m^− 2^) according to surgical positioning

Surgical position	HR	IBP			NIBP			CS-BP			CS-CI
		sys	MAP	dia	sys	MAP	dia	sys	MAP	dia	
Supine	77.6 (± 15.8)	129.8 (± 26.9)	87.9 (± 19.2)	65.4 (± 13.0)	129.8 (± 27.5)	86.3 (± 16.6)	72.4 (± 14.5)	123.1 (± 28.9)	91.8 (± 21.0)	72.7 (± 15.7)	2.4 (± 0.8)
Beach chair	73.7 (± 14.6)	115.1 (± 26.4)	76.7 (± 19.4)	57.8 (± 14.3)	114.6 (± 24.8)	76.6 (± 13.9)	64.6 (± 11.2)	102.1 (± 21.3)	76.5 (± 13.5)	61.6 (± 9.4)	2.3 (± 0.7)
Combined (supine and beach chair)	75.6 (± 15.3)	120.9 (± 27.5)	81.2 (± 20.1)	60.9 (± 14.3)	122.4 (± 27.3)	81.6 (± 16.1)	68.6 (± 13.6)	112.7 (± 27.5)	84.3 (± 19.3)	67.2 (± 14.1)	2.4 (± 0.7)

*HR* Heart rate, *IBP* Invasive blood pressure, *NIBP* Non-invasive blood pressure, *CS-BP* ClearSight™ blood pressure, *CS-CI* ClearSight™ cardiac index, *sys* Systolic, *MAP* Mean arterial pressure, *dia* Diastolic

**Fig. 2:**
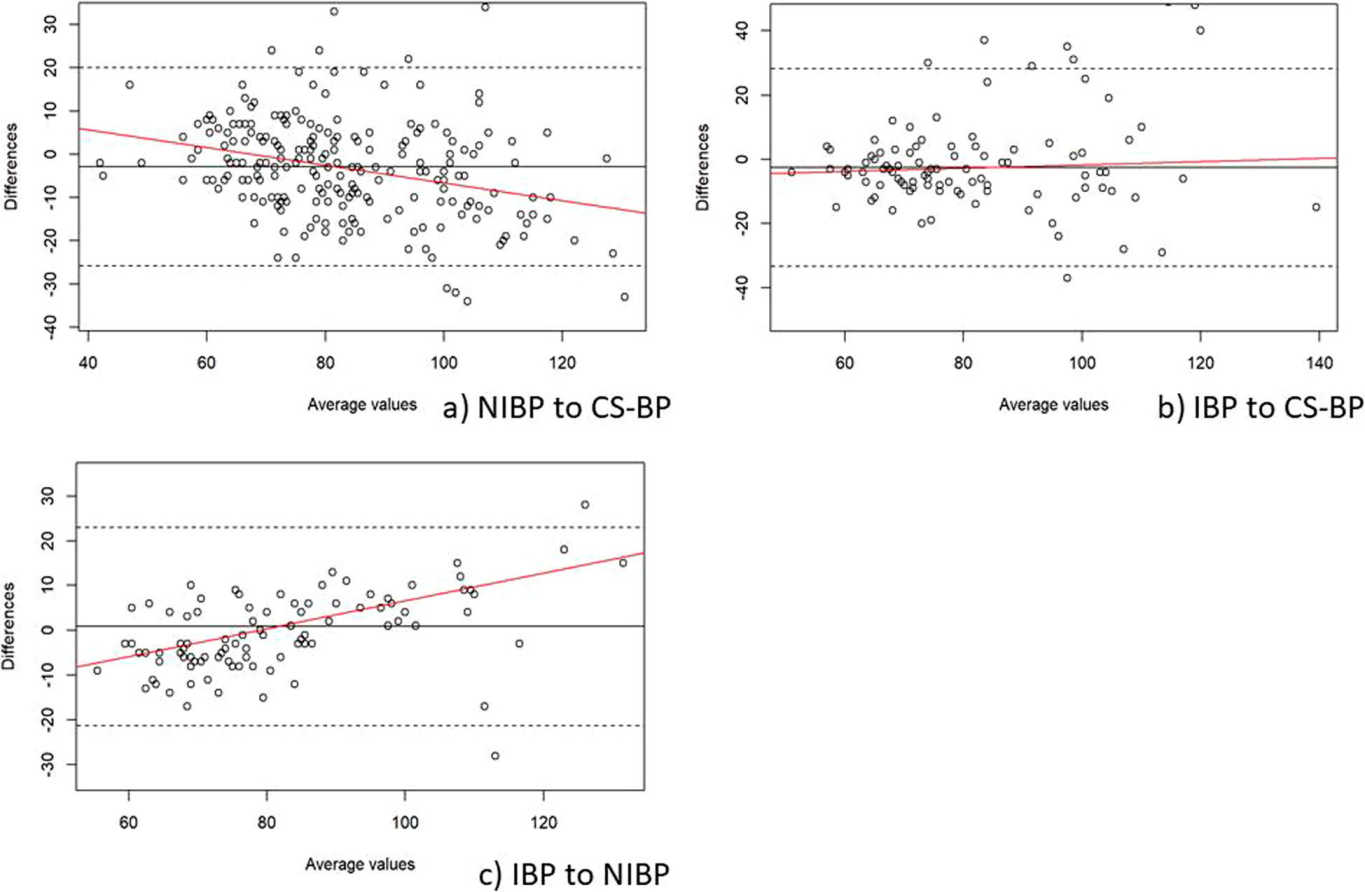
Bland-Altman plots for the overall comparison of mean arterial blood pressure (MAP; mmHg) obtained from (**a**) intermittent non-invasive oscillometric blood pressure assessment (NIBP) to ClearSight™ blood pressure (CS-BP), (**b**) invasive blood pressure assessment (IBP) to CS-BP, and (**c**) IBP to NIBP. The solid lines illustrate the mean difference and the dashed lines indicate average differences ±1.96 standard deviation of the difference. The red lines visualize the regression line and indicate whether the differences are dependent on the size of MAP values

The MAP Bland-Altman analyses showed a mean of the differences (± SD; 95% limits of agreement) between CS-BP and NIBP of − 2.9 mmHg (± 11.7; − 25.8 - 20.1), between CS-BP and IBP of − 2.6 mmHg (± 15.7; − 33.4 - 28.2), and between NIBP and IBP of 0.9 mmHg (± 11.3; − 21.3 – 23.0) (Fig. 2).

**Fig. 3:**
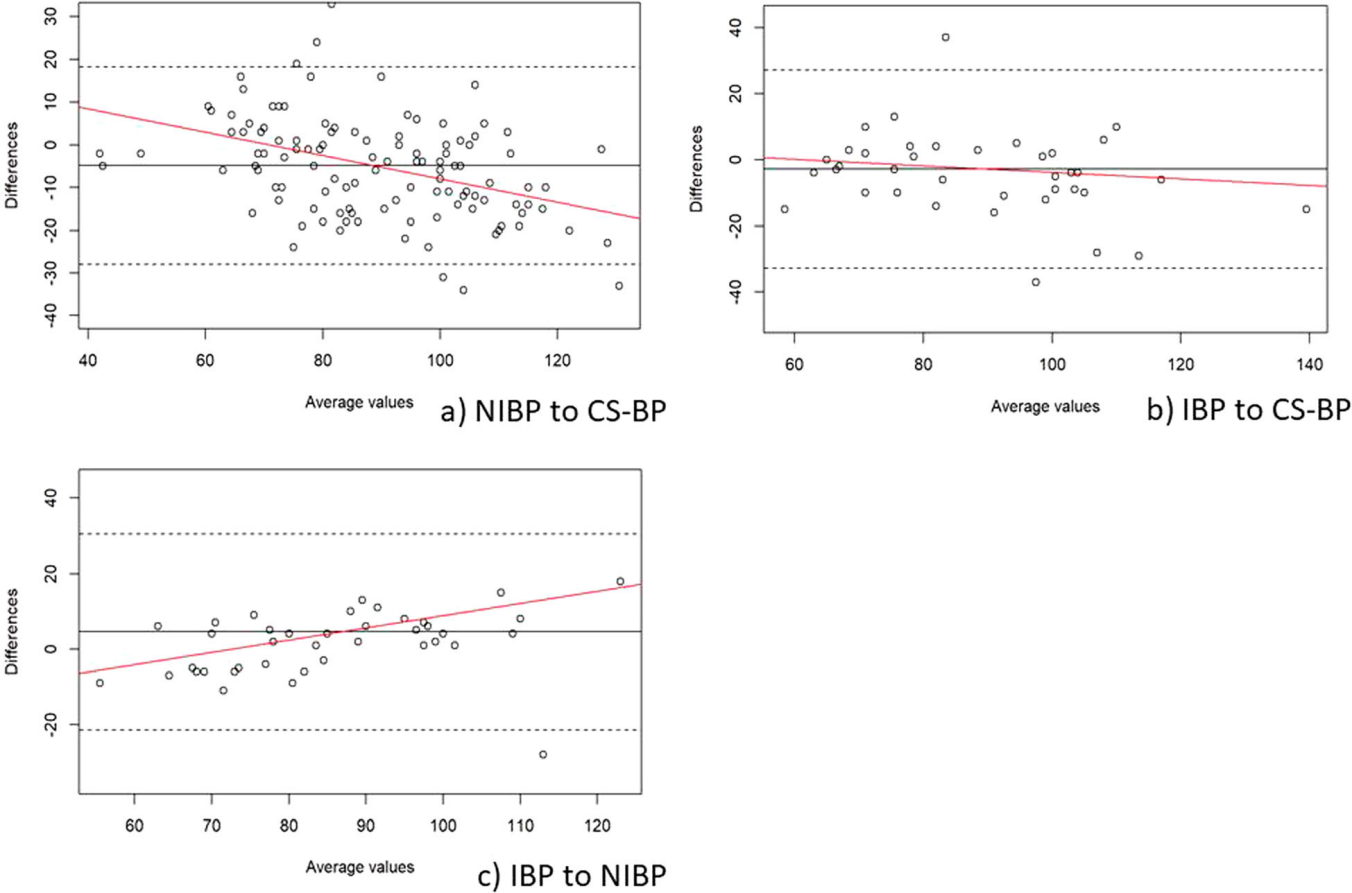
Bland-Altman plots for the comparison during supine positioning of mean arterial blood pressure (MAP; mmHg) obtained from (**a**) intermittent non-invasive oscillometric blood pressure assessment (NIBP) to ClearSightTM blood pressure (CS-BP), (**b**) invasive blood pressure assessment (IBP) to CS-BP, and (**c**) IBP to NIBP. The solid lines illustrate the mean difference and the dashed lines indicate average differences ±1.96 standard deviation of the difference. The red lines visualize the regression line and indicate whether the differences are dependent on the size of MAP values

**Fig. 4:**
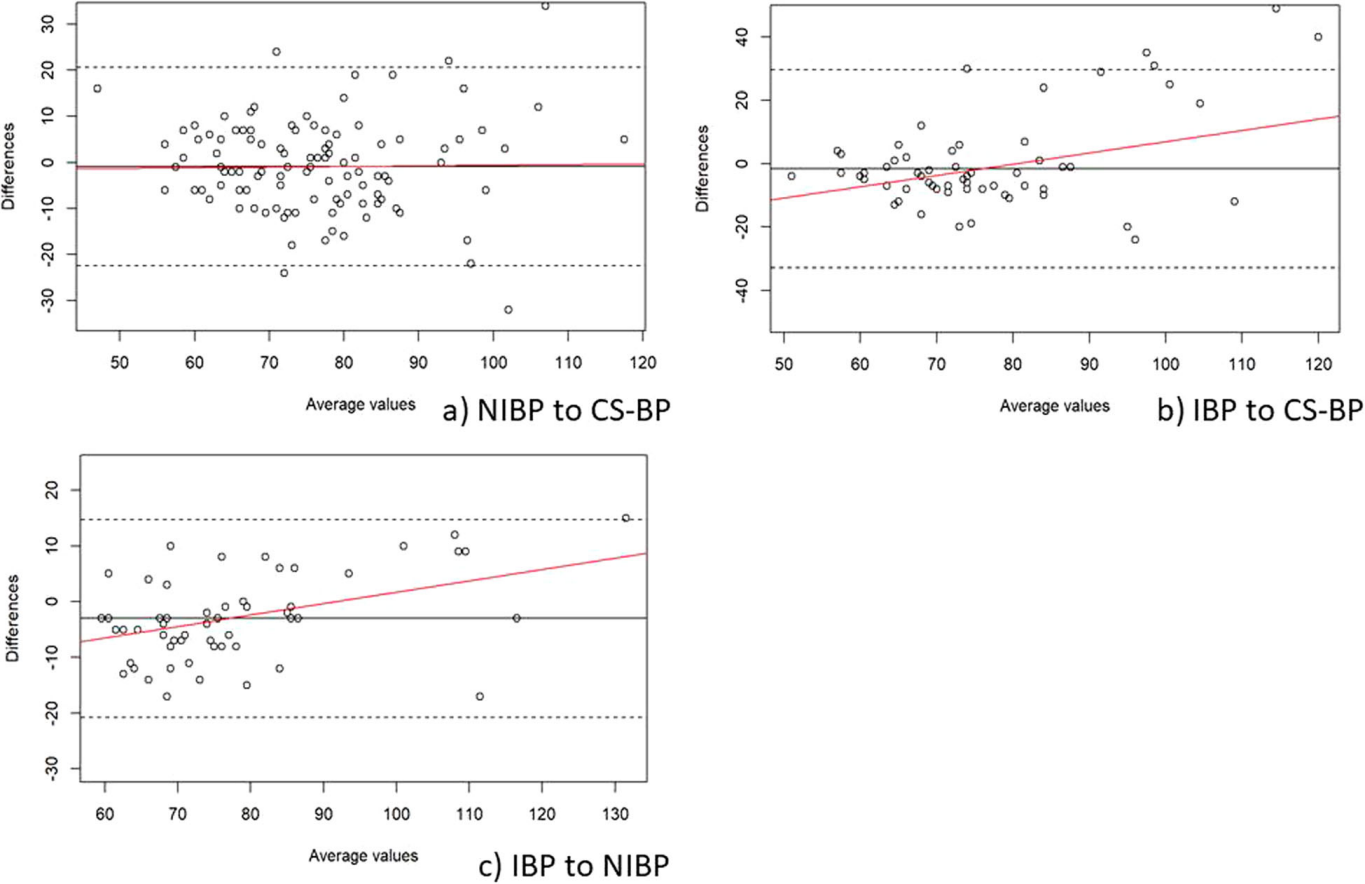
Bland-Altman plots for the comparison during beach chair positioning of mean arterial blood pressure (MAP; mmHg) obtained from (**a**) intermittent non-invasive oscillometric blood pressure assessment (NIBP) to ClearSightTM blood pressure (CS-BP), (**b**) invasive blood pressure assessment (IBP) to CS-BP, and (**c**) IBP to NIBP. The solid lines illustrate the mean difference and the dashed lines indicate average differences ±1.96 standard deviation of the difference. The red lines visualize the regression line and indicate whether the differences are dependent on the size of MAP values

In the supine position, the MAP Bland-Altman analyses showed a mean of the differences (± SD; 95% limits of agreement) between CS-BP and NIBP of − 4.9 mmHg (± 11.8; − 28.0 – 18.2), between CS-BP and IBP of − 2.8 mmHg (± 15.3; − 32.8 – 27.1), and between NIBP and IBP of 4.6 mmHg (± 13.3; − 21.4 – 30.6) (Fig. 3). In the beach chair position, the corresponding values for MAP from Bland-Altman analysis for the mean of the differences (± SD; 95% limits of agreement) were − 0.9 mmHg (± 11.0; − 24.0 – 22.2) between CS-BP and NIBP, − 1.6 mmHg (± 16.0; − 32.9 – 29.7) between CS-BP and IBP, and − 3.0 mmHg (± 9.1; − 20.8 – 14.7) between NIBP and IBP (Fig. 4). Results from the weighted one-sample t-tests are shown in Table 3.

**Table 3:** Results of statistical testing comparing MAP blood pressure assessment methods according to surgical position

Surgical position	NIBP to CS-BP	IBP to CS-BP	IBP to NIBP
Supine	*P* < 0.001 *N* = 116	*P* = 0.245 *N* = 40	*P* = 0.034 *N* = 40
Beach chair	*P* = 0.348 *N* = 110	*P* = 0.344 *N* = 59	*P* = 0.007 *N* = 58
Combined (supine and beach chair)	*P* < 0.001 *N* = 226	*P* = 0.058 *N* = 99	*P* = 0.429 *N* = 98

*MAP* Mean arterial pressure, *IBP* Invasive blood pressure, *NIBP* Non-invasive blood pressure, *CS-BP* ClearSight™ blood pressure. *P*-values calculated using weighted one-sample t-test

The respective values for systolic and diastolic blood pressure are provided in Table 4.

**Table 4:** Mean of the differences (mmHg; ± SD; 95% limits of agreement) using Bland-Altman analyses for the comparison of blood pressure assessment methods

Surgical position	NIBP to CS-BP			IBP to CS-BP			IBP to NIBP		
	sys	MAP	dia	Sys	MAP	dia	sys	MAP	dia
Supine	7.3 (± 17.5;-27.1–41.7)	−4.7 (± 11.8;-28.0–18.2)	−0.4 (± 10.2;-20.3–19.6)	10.5 (± 19.5;-27.8–48.7)	−2.8 (± 15.3;-32.8–27.1)	−6.8 (± 13.3;-32.9–19.3)	0.6 (± 15.0;-28.8–29.9)	4.6 (± 13.3;-21.4–30.6)	−4.4 (± 11.9;-27.6–18.9)
Beach chair	9.8 (± 21.4;-32.1–51.7)	−0.9 (± 11.0;-24.0–22.2)	2.1 (± 8.6;-14.7–19.0)	10.4 (± 21.9;-32.5–53.2)	−1.6 (± 16.0;-32.9–29.7)	−5.0 (± 10.1;-24.7–14.8)	−7.3 (± 10.4;-27.7–13.1)	−3.0 (± 9.1;-20.8–14.7)	−7.8 (± 7.7;-22.8–7.3)
Combined	8.5 (± 19.6; − 30.1 – 47.0)	− 2.9 (± 11.7;-25.8–20.1)	0.9 (± 9.6; − 17.8 – 19.7)	10.4 (± 21.7; − 32.1 – 52.9)	− 2.6 (± 15.7;-33.4–28.2)	−6.5 (± 11.5; − 29.0 – 16.1)	−4.2 (± 12.8; − 29.3 – 21.0)	0.9 (± 11.3; − 21.3 – 23.0)	− 5.9 (± 9.6; − 24.8 – 13.0)

*IBP* Invasive blood pressure, *NIBP* Non-invasive blood pressure, *CS-BP* ClearSight™ blood pressure, *Sys* Systolic, *MAP* Mean arterial pressure, *dia* diastolic

Spearman’s rank correlation of differences in MAP values for CS-BP compared to NIBP (in both supine and beach chair positions) showed a weak but significant negative correlation to CS-CI (rho = − 0.287; *p* < 0.0001), but not to heart rate (rho = − 0.061; *p* = 0.388). When compared to IBP, there was also a significant negative correlation to cardiac index (rho = − 0.245; *p* = 0.017), but not to heart rate (rho = − 0.042; *p* = 0.683).

Overall, 49.6% (112/226) MAP comparisons indicated a ≥ 10% deviation between CS-BP and NIBP measurements. The corresponding number for CS-BP MAP compared to IBP was 46.5% (46/99). 35.7% (35/98) MAP comparisons differed ≥10% between NIBP and IBP measurements.

For consecutive MAP values of NIBP and IBP, an increase or decrease of ≥10% was accompanied by a change in CS-BP MAP in the same direction in 94% (79/84) compared to NIBP, and 95.3% (41/43) compared to IBP. When there was an increase or decrease of ≥10% in IBP, it was accompanied by a change in NIBP in the same direction in 90.7% (39/43).

## Discussion

In this study, a continuous non-invasive blood pressure monitoring system (ClearSight™) was compared with oscillometric and invasive blood pressure monitoring in patients undergoing shoulder surgery in the beach chair position. Our main finding was that the accuracy of the ClearSight™ blood pressure readings was not worse in the beach chair position than in the supine position.

ClearSight™ and other similar systems were validated using invasive reference methods in different clinical settings, which led to varying and contradictory results [10, 18, 20]. Performance for mean arterial pressure was better than systolic values [15, 21]. This can be explained by physiological differences between the assessment site and the subsequently reconstructed brachial reconstructed brachial arterial pressure estimation. In our study, we also found that the differences mainly in the systolic values resulted in larger limits of agreement than would be desirable for clinical decision making.

During elective orthopedic surgery with patients in the supine position, Balzer et al. found a tendency to higher precision compared to IBP for the volume clamp method than for NIBP [18]; however, the correlation between IBP and the tested device (Nexfin; Edwards Life Sciences, Irvine, USA) was lower than reported during cardiac surgery. The authors contributed the variation to the more static patient positioning during cardiac versus orthopedic surgery [18]. In our study, the patient positioning may have been even less static given that patients were brought to the beach chair position, and the operated arm was moved relatively often during the shoulder surgery. These two factors may explain, in part, differences between CS-BP and standard monitoring found in our study population.

The Association for the Advancement of Medical Instrumentation (standards for non-invasive arterial pressure measurement) defines 5 mmHg (± 8 mmHg) as a clinically acceptable agreement level when comparing a test to a reference method [10]. However, this may be overly optimistic. According to large observational studies comparing oscillometric non-invasive to invasive arterial blood pressure measurements, MAP differences between methods were as high as 10 mmHg [22]. A meta-analysis comparing continuous non-invasive blood pressure measurements using the volume clamp method (amongst others Nexfin; now Edwards Life Sciences, Irvine, USA) to invasive MAP found a bias of 3.9 mmHg (± 8.7) [23]. In our patient population, the mean of the difference was below 5 mmHg for all blood pressure modalities in supine and beach chair position, but the standard deviation regularly exceeded ±8 mmHg. Furthermore, the 95% limits of agreement shown by Bland-Altman comparisons were continuously larger than what would be considered an acceptable deviation from the “real” blood pressure in clinical anesthesia.

In more than 90% of the time during our investigation, the ClearSight™ system detected changes in the arterial blood pressure of 10 % or higher. Furthermore, the system’s performance was not worse than intermittent standard blood pressure monitoring when compared to invasive arterial blood pressure assessment. However, there is a greater potential for bias when interpreting intermittent versus continuous monitoring. The interpretation of trends rather than absolute values may be more useful with this new system.

Interestingly, there was a negative correlation between the MAP differences and the cardiac index (i.e., more substantial differences with lower cardiac index). One possible explanation was that signal quality was less optimal with lower cardiac indices. However, this remains speculative.

Our study had limitations. First, we included a relatively small number of patients, and we used invasive blood pressure measurements as a reference method for only half of the cohort. We chose to limit the use of invasive technique to only those who needed additional arterial blood pressure monitoring because we did not want to expose the other patients to unnecessary risk of complications, such as infection. As a result of the small sample size, we observed wide standard deviations. Second, we were obligated to keep the blood pressure values in a narrow range due to clinical standards for orthopedic surgeries, which prevented us from performing an error grid analysis as proposed by Saugel et al. [24] Third, given the mainly descriptive nature of this study and the lack of published data on patients in beach chair positioning, we did not do a power calculation, but with 100 data points, a comparison of methods via Bland-Altman plots for supine and beach chair position seemed feasible [19]. Fourth, it would have been preferable to set up the devices on different arms, especially as performing NIBP measurements induces a state of hypoperfusion that affects invasive blood pressure measurement and readings by the volume clamp method. However, given that only one arm is available for all installations during shoulder surgery, we recorded the values of the continuous measurement methods just before starting NIBP measurements. Fifth, as we do under regular clinical practice, we proceeded with general anesthesia just after applying the interscalene blocks without formal assessment of the block’s performance. Therefore, we were not able to evaluate the influence of the block on hemodynamics or our findings. Sixth, we speculate that the accuracy of the volume clamp method could be affected by vascular tone and overall blood volume [23]. We did not record the exact use of vasopressors (although patients only received ephedrine, if any) or the volume of fluid administered versus blood loss during surgery. Clinical practice, however, often functions without precise information on the correlation between blood pressure measurements and the actual fluid balance. Lastly, both the invasively measured and the ClearSight™ arterial blood pressure were zeroed to the ambiance at the level of the NIBP cuff, which was at the upper arm. Calibration of the pressure transducers, which was not repeated for both supine and beach chair positions in our study, is more commonly done at the level of the right atrium. This could have resulted in an underestimation of the measurement differences. However, the calibration level at the proximal end of the NIBP cuff comes close to the level of the right atrium, even in the beach chair position.

## Conclusions

The levels of agreement among the three blood pressure measurements methods (non-invasive intermittent, non-invasive continuous, and invasive continuous) were comparable between the beach chair and supine positions. Non-invasive continuous blood pressure monitoring with the volume clamp method has the potential to bridge the gap between non-invasive intermittent oscillometric monitoring and continuous invasive blood pressure monitoring.

## Data Availability

The datasets used and/or analyzed during the current study are available from the corresponding author on reasonable request.

## Abbreviations

ABP: Arterial blood pressure
ASA: American Society of Anesthesiologists physical status
BMI: Body mass index
CS-BP: ClearSight™ blood pressure
CS-CI: ClearSight™ cardiac index
dia: Diastolic
HR: Heart rate
IBP: Invasive blood pressure
IQR: Interquartile range
MAP: Mean arterial pressure
NIBP: Non-invasive blood pressure
SD: Standard deviation
sys: Systolic
